# Effectiveness of glucocorticoids in preventing esophageal stricture and predictors of stricture after esophageal ESD: 5 years of experience in a single medical center

**DOI:** 10.3389/fmed.2025.1428508

**Published:** 2025-02-25

**Authors:** Qingxia Wang, Yuan Ding, Qiliu Qian, Yinnan Zhu, Ruihua Shi

**Affiliations:** Department of Gastroenterology, Medical School, Southeast University Affiliated Zhongda Hospital, Nanjing, China

**Keywords:** endoscopic mucosal resection, esophageal stenosis, hyaluronic acid, steroids, risk factors

## Abstract

**Background:**

Esophageal stricture is one of the major complications after endoscopic submucosal dissection (ESD) of the esophagus. However, even with steroid prophylaxis, stenosis still occurs in up to 45% of patients. Accordingly, the aim of this study was to evaluate the efficacy and safety of steroid therapy in preventing esophageal strictures after ESD, as well as to assess the predictors of esophageal strictures after the application of steroids.

**Methods:**

Between February 2018 and March 2023, 207 patients who underwent esophageal ESD at Southeast University Affiliated Zhongda Hospital were retrospectively enrolled. We evaluated stenosis rate, number of endoscopic dilations after ESD, the interval between the first endoscopic dilatation after ESD and explored risk factors for strictures after steroid prophylaxis.

**Results:**

In the control group, the oral steroids group, and the combined group, the stenosis rates were 83/87 (95.4%), 44/53 (83.0%), and 56/67 (83.6%), respectively; the number of endoscopic dilations were 3.43 (±2.22), 2.34 (±2.17), and 1.52 (±1.25), respectively; the time intervals between first endoscopic dilation and ESD procedure were 38.36 (±6.87), 68.18 (±9.49), and 96.82 (±8.41) days, respectively; all these indicators were significantly better in the oral and combined groups than in the control group (*p* < 0.05). Multivariate analysis identified lesion circumference ≥ 5/6th and submucosal injection of solution were two independent factors on esophageal stricture formation (*p* < 0.05).

**Conclusion:**

Steroid prophylaxis is effective and safe in preventing esophageal stenosis. Moreover, lesion circumference and submucosal injection of sodium hyaluronate were two independent factors on esophageal stricture formation even with steroids administration.

## Introduction

Esophageal cancer is the seventh most common type of cancer worldwide and constitutes the sixth leading cause of cancer deaths (1). However, the 5-year survival rate for patients who undergo endoscopic resection for esophageal cancer at an early stage now exceeds 90% (2). Endoscopic submucosal dissection (ESD) has been considered as a prominent method for early esophageal cancer resection (3, 4). Although ESD has the advantages of overall tumor resection, more accurate histological diagnosis, and reduced risk of local recurrence (3), there are also concerns about postoperative esophageal stenosis that requires attention (5). Some studies have reported a high risk of postoperative stricture with ESD resections greater than 3/4 of the circumferential diameter, especially for total circumferential resections, with esophageal stricture rates reaching 100% (6–8). Patients with esophageal strictures after ESD usually require multiple endoscopic balloon dilatations(EBDs) or probe strip dilatation for symptomatic relief, which severely affects quality of life and increases healthcare costs (9, 10).

As a result, researchers have devised various methods to prevent stricture formation in the esophagus following ESD, including medications, mechanical devices, tissue engineering, and autologous tissues (11–13). Among these strategies, steroid prophylaxis of esophageal strictures, especially local injection of triamcinolone acetonide (TA), is currently a relatively effective method of preventing esophageal strictures (14–16). However, even after local TA injection, stenosis occurs in up to 45% of patients with non-circumferential resection (14, 17). Therefore predicting the risk of stenosis formation after local TA injection is essential because it allows additional interventions in risky patients. Nevertheless, few studies have investigated the predictors of stenosis formation after local TA injection.

Thus, the purpose of this retrospective study was mainly to evaluate the efficacy and safety of steroids prophylaxis in preventing post-ESD esophageal stenosis, as well as to assess the predictors of esophageal strictures after steroid application.

## Materials and methods

### Patients

**Figure 1:**
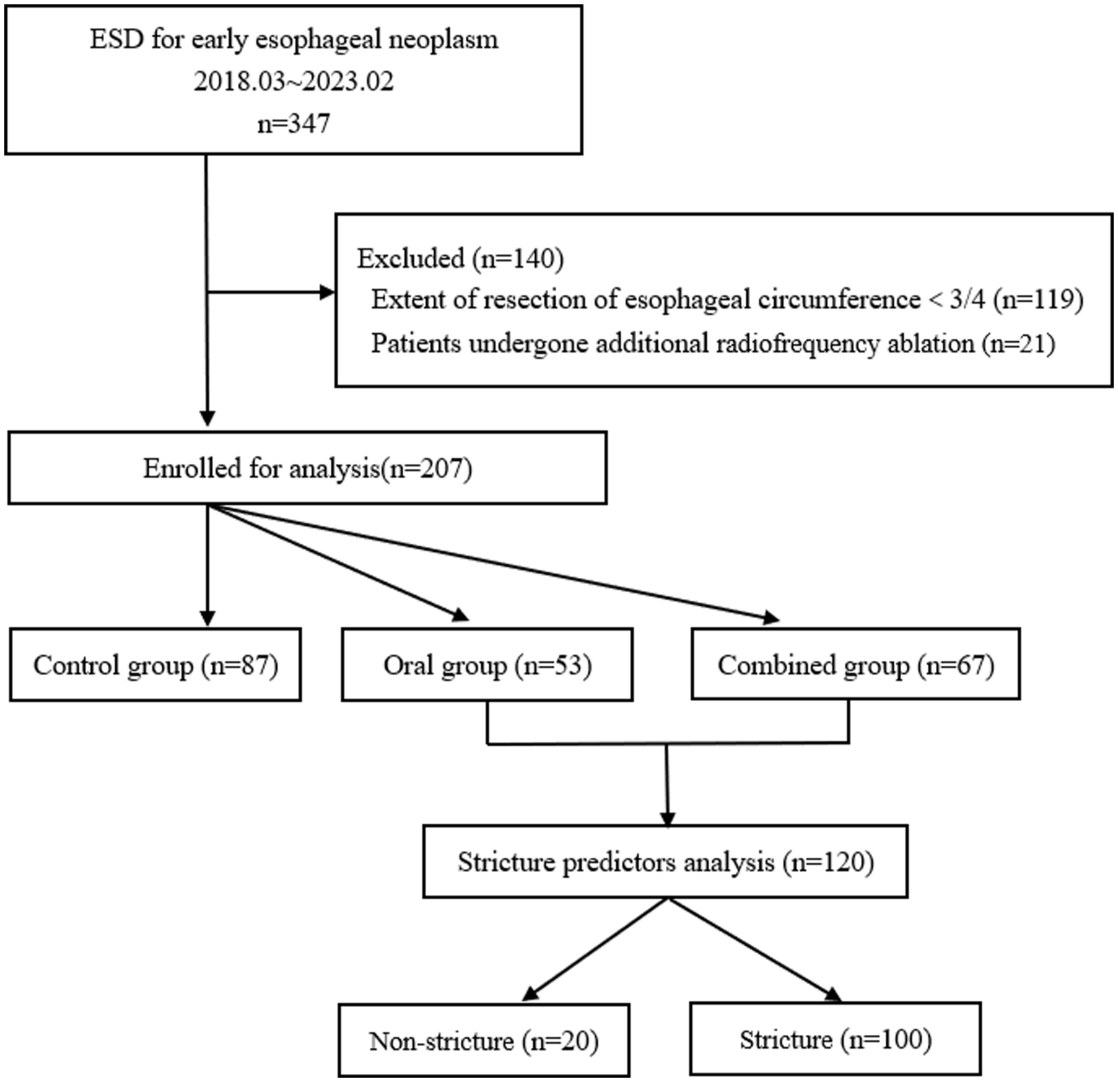
Flow diagram of the study.

Patients with superficial esophageal cancers who underwent ESD at Southeast University Affiliated Zhongda Hospital between February 2018 and March 2023 were included in this study (Figure 1).

The inclusion criteria were as follows: (i) patients with superficial esophageal carcinoma which was indications for ESD; (ii) mucosal defects ≥3/4 of the circumferential esophageal lumen following ESD and (iii) histologically confirmed high-grade squamous intraepithelial neoplasia or squamous cell carcinoma after ESD.

The exclusion criteria were as follows: (i) preoperative pathology suggestive of poorly differentiated or undifferentiated carcinoma; (ii) history of esophageal surgery and radiotherapy; (iii) additional surgical treatment or radiofrequency ablation required after ESD; (iv) inability to follow up for more than 6 months; and (v) long-term use of glucocorticoids.

This study was approved by the Southeast University Affiliated Zhongda Hospital Ethics Committee approval (2018ZDSYLL018-P01), and all patients were informed and signed an informed consent form.

### ESD procedure

All procedures were performed by experienced endoscopists at our center who had been working for at least 5 years and had performed more than 100 ESD esophageal procedures prior to this study. All operations were performed under general anesthesia with tracheal intubation. Tip-covered knife (IT knife, KD-611 L; Olympus), tip-uncovered knife (Dual knife KD-650 Q, Olympus), hook knife (KD-620LR, Olympus), or hybrid knife (Erbe Elektromedizin GmbH) were used in endoscopic submucosal dissection. Intraoperative bleeding was performed using an electrocoagulation (FD-410 LR, Olympus); single-channel endoscopes with hoods (GIFQ 260, GIF-Q260 J, Olympus) were used for endoscopy; and endoscopic electrosurgical generator ESG-100 (Olympus) was used for ESD procedures. A 3% Lugol solution was used to clarify the margins of the lesion, and a needle knife or a double knife was used to mark 2 mm outside the margins of the target lesion. Two submucosal injections were used: primarily epinephrine glycerol solution and diluted indigo carmine or melphalan, while diluted hyaluronic acid was used when submucosal fibrosis was encountered. Electrocoagulation modes were Endo Cut I, forced coagulation, or rapid coagulation mode. The endoscopist retreated the scope after spraying fibrin glue on some wounds based on experience.

### Treatment strategy to prevent post-ESD strictures

In the control group, patients had no postoperative stenosis prevention measures. In the oral group, 8-week therapy was adopted, i.e., oral prednisone acetate 30 mg/d was started on the 3rd day after ESD surgery, then reduced to 25 mg/d after 2 weeks, then to 20 mg/d after 2 weeks, then to 15 mg/d after 1 week, then to 10 mg/d after 1 week, then to 5 mg/d after 1 week, and then discontinued on the 9th week. In the oral combined with local injection group, tretinoin injection 80 mg was given to the residual submucosal layer of the lesion during the ESD surgery, and 8 weeks of oral prednisone therapy (same as the oral group) was started on the 3rd day of the postoperative period. Proton pump inhibitors were routinely administered orally after ESD in the control group, the oral group, and the oral combined with local injection group.

### Follow-up and outcomes

Gastroscopy was performed 3 months after ESD, and endoscopic dilatation of the exploratory strip was performed at any time when the patient developed symptoms of dysphagia during the period. The follow-up period was up to March, 2023.

**Figure 2:**
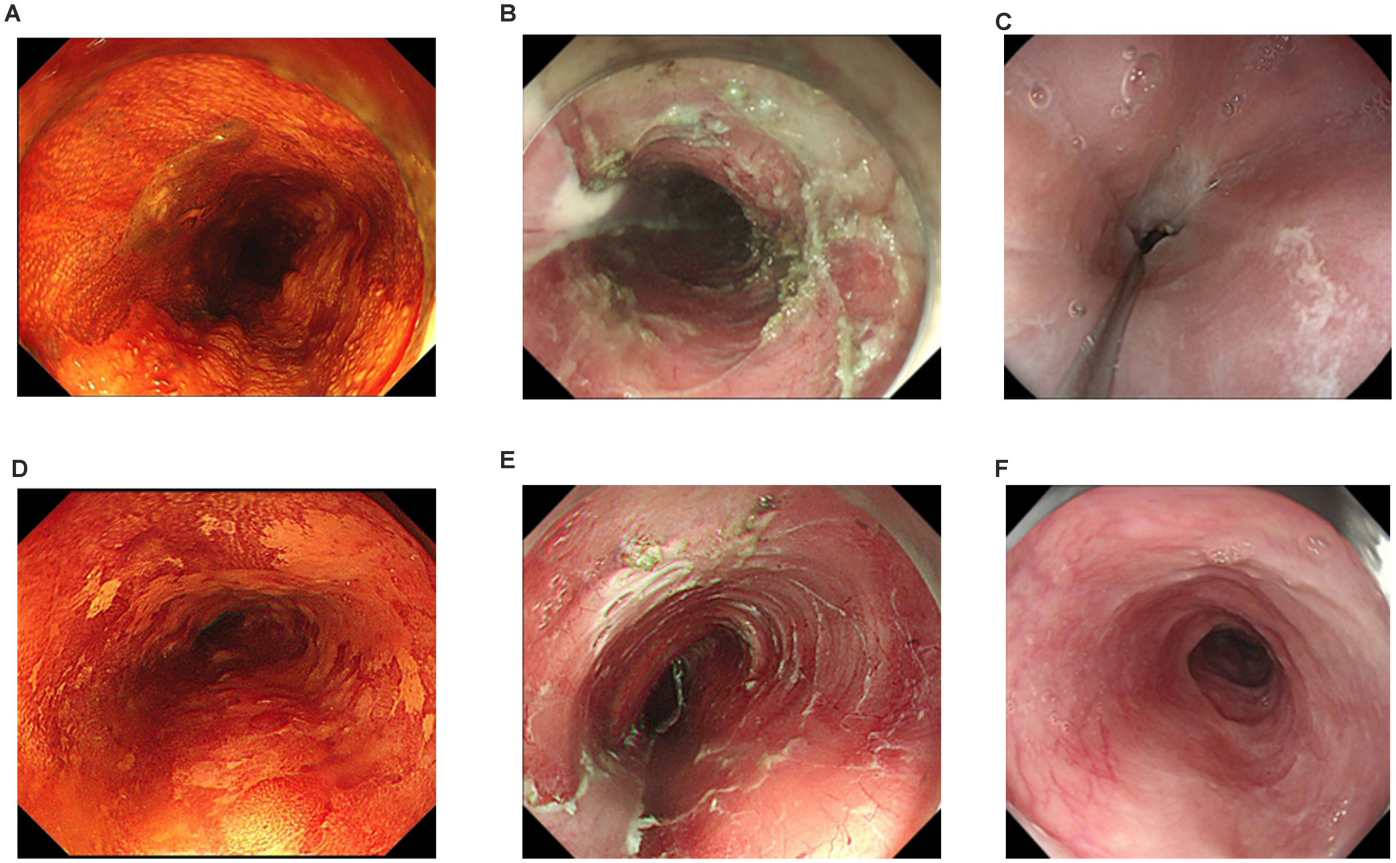
**(A)** Lugol’s chromoendoscopy showed lesion located at 18–24 cm of the incisors; **(B)** The mucosal defect after ESD involved 4/5th of the esophageal circumference and the longitudinal length of defect was 65 mm. The patient took no prophylactic measures; **(C)** Stricture developed after 1 month located at 20 cm of the incisors. The patient underwent a total of three exploratory strip dilatations after esophageal stricture; **(D)** Lugol’s chromoendoscopy showed lesion located at 28–35 cm of the incisors; **(E)** The mucosal defect after ESD involved 4/5th of the esophageal circumference and the longitudinal length of defect was 55 mm. This patient took prophylactic measures to prevent postoperative stenosis with local multipoint injections of triamcinolone acetonide and oral prednisone; **(F)** No stricture developed after 7 months. The standard diagnostic endoscope (9.8 mm in diameter) could pass through the ESD wound scar smoothly.

The outcome data mainly included: (1) effectiveness indicators: stenosis rate, number of endoscopic dilations after ESD, and the interval between the first endoscopic dilatation after ESD; (2) relevant predictors were collected: age, gender, body mass index(BMI), smoke, characteristics of lesions (tumor location, longitudinal length of lesion, macroscopic type, tumor invasion depth, histopathologic diagnosis), additional chemoradiotherapy (CRT), type of endo-knife, procedure time, Electrosurgical unit modes, type of submucosal injection solution, en bloc resection, fibrin glue; (3) safety indicators: whether there were surgical and glucocorticoid-related adverse events, as well as the adverse reactions of endoscopic dilatation. Stenosis was defined as diameter of stricture section below 9.8 mm which a standard endoscope (GIF H 260, Olympus) could not pass through (Figure 2). En bloc resection was defined as removal of the lesion as a single specimen.

### Statistical analysis

Categorical variables are presented as counts and percentages. The continuous and normally distributed variables are presented as the mean ± SD. The *χ*2 test was performed to compare categorical variables while *T* test was employed for comparisons of continuous variables. Univariate and multivariate regression analyses were conducted to detect the predictors for stricture in patients with steroids prophylaxis. *p* value <0.05 was considered to indicate a statistically significant difference. Statistical analysis was performed by using JMP Pro software version 16.0 (SAS Institute, Inc., Cary, NC, USA).

## Results

### Patient flow and characteristics

Characteristics of all patients were presented in Table 1. From February 2018 to March 2023, a total of 207 patients were finally included in this study, with a mean age of 67 years, 141 (68.1%) patients were male, and 101 (48.8%) lesions were located in the mid-thoracic esophagus. There were 87 patients in the control group, 53 patients in the oral steroids group, and 67 patients in the oral steroids combined with topical injection of triamcinolone acetonide group. There were no statistically significant differences between the 3 groups of patients in most baseline data, but there were statistically significant differences in lesion circumference, lesion length, and lesion depth.

**Table 1:** Demographics and characteristics of patients included in the study.

Variables	Control group (*n* = 87)	Oral group (*n* = 53)	Combined group (*n* = 67)	*F*/χ2	*p*
Age (years, x̄ ± *s*)	67.28 ± 8.34	67.70 ± 6.87	66.49 ± 8.29	0.37	0.69
BMI (kg/m2, x̄ ± *s*)	22.62 ± 2.82	22.87 ± 3.23	22.77 ± 3.00	0.13	0.88
Gender (male, %)	57 (65.5)	33 (62.3)	51 (76.1)	3.16	0.21
Smoke (%)	21 (24.1)	13 (24.5)	15 (22.4)	0.09	0.95
Macroscopic type (*n* [%])				3.63	0.46
0-IIa	17 (19.5)	11 (20.7)	9 (13.4)		
0-IIb	55 (63.2)	32 (60.4)	39 (58.2)		
0-IIc	15 (17.2)	10 (18.9)	19 (28.4)		
Histopathologic diagnosis (*n* [%])				3.0	0.22
High-grade intraepithelial neoplasia	46 (52.9)	25 (47.2)	26 (38.8)		
Esophageal squamous cell carcinoma	41 (47.1)	28 (52.8)	41 (61.2)		
Lesion circumference (*n* [%])				29.74	<0.01
<5/6 and ≥ 3/4	80 (92.0)	37 (69.8)	37 (55.2)		
≥5/6	7 (8.0)	16 (30.2)	30 (44.8)		
Length of lesion (mm, x̄ ± *s*)	44.52 ± 2.04	61.36 ± 2.61	60.55 ± 2.32	18.76	<0.01
Depth of invasion				13.16	0.01
M1/M2	76 (87.4)	40 (75.5)	49 (73.1)		
M3/SM1	3 (3.5)	8 (15.1)	14 (20.9)		
SM2	8 (9.2)	5 (9.4)	4 (6.0)		
Additional chemoradiotherapy (%)	14 (16.1)	7 (13.2)	10 (14.9)	0.22	0.90
Tumor location (*n* [%])				0.01	0.58
Cervical and upper thoracic esophagus	26 (29.9)	13 (24.5)	13 (19.4)		
Middle thoracic esophagus	41 (47.1)	24 (45.3)	36 (53.7)		
Lower thoracic esophagus	20 (23.0)	16 (30.2)	18 (26.9)		

M1, intraepithelial; M2, lamina propria; M3, muscularis mucosae; SM1 < 200 μm from the muscularis mucosae; SM2 ≥ 200 μm from the muscularis mucosae.

### Stricture rate

The stricture rate was 83/87 (95.4%) in the control group was, 44/53 (83.0%) in the oral steroids group, and 56/67 (83.6%) in the oral steroids combined with local injection group, a statistically significant difference (*χ*^2^ = 7.95, *p* = 0.02). Further comparison between the two groups revealed that the stenosis rate was lower in the oral group than in the control group (*χ*^2^ = 5.81, *p* = 0.02), and also lower in the combined group (*χ*^2^ = 6.07, *p* = 0.01). Whereas, there was no statistically significant difference between the combined and oral groups (*χ*^2^ = 0.01, *p* = 0.93).

### The number of endoscopic dilations after ESD

The number of endoscopic dilations was 3.43 (±2.22) in the control group, 2.34 (±2.17) in the oral group, and 1.52 (±1.25) in the combined group, with a statistically significant difference between the 3 groups (*F* = 18.46, *p* < 0.01). Further comparisons between the two groups showed that the oral group was able to significantly reduce the number of endoscopic dilations after ESD compared to the control group (*F* = 6.27, *p* = 0.01), and the combined group was also able to significantly reduce the number of endoscopic dilations compared to the control group (*F* = 39.46, *p* < 0.01). When compared to the oral group, the combined group could reduce the number of endoscopic dilations as well (*F* = 6.73, *p* = 0.01).

### Time interval between first endoscopic dilation and ESD procedure

The time interval between first endoscopic dilation and ESD procedure was 38.36 (±6.87) days in the control group, 68.18 (±9.49) days in the oral group, and 96.82 (±8.41) days in the combined group, with a statistically significant difference between the 3 groups (*F* = 14.67, *p* < 0.01).

Further comparison between the two groups suggested significantly longer intervals in the oral group than in the control group (*F* = 6.85, *p* = 0.01); the intervals were also significantly longer in the combined group than in the control group (*F* = 37.18, *p* < 0.01). Simultaneously, the interval was also significantly longer in the combined group than in the oral group (*F* = 3.56, *p* = 0.03).

### Potential factors associated with esophageal strictures after ESD with steroids prophylaxis

Stenosis-influencing factors were analyzed in 120 patients in whom glucocorticoids were used for stenosis prophylaxis, either oral prednisone or oral prednisone combined with local tretinoin injection. Among them, univariate analysis showed lesion circumferential size and submucosal injection solution as significant correlates with the risk of stenosis formation. Logistic regression analyses were then performed using predictors that were significant in univariate analyses and combined with known predictors from previous reports (17–19). We found that lesion circumference and submucosal injection solution were two independent factors on esophageal stricture formation. Stricture rates stratified according to predictors are shown in Table 2.

**Table 2:** Univariate and multivariate logistic regression analyses of predictors of post-esophageal ESD stricture formation.

Characteristics	Univariate analysis			Multivariate analysis	
	Non-stricture (*n* = 20)	Stricture (*n* = 100)	*p* value	OR (95% CI)	*p* value
Age	66.70 ± 10.41	67.09 ± 7.09	0.84	-	-
Gender			0.12		
Male	11 (55.0%)	129 (70.5%)			
Female	9 (45.0%)	54 (29.5%)			
BMI	22.81 ± 3.72	22.82 ± 2.97	0.99	-	-
Smoke			0.31		
Yes	3 (15.0%)	25 (25.0%)		-	-
No	17 (85.0%)	75 (75.0%)		-	-
Histopathologic diagnosis			0.21		
High-grade intraepithelial neoplasia	6 (30.0%)	45 (45.0%)		-	-
Esophageal squamous cell carcinoma	14 (70.0%)	55 (55.0%)		-	-
Additional chemoradiotherapy			0.54		
Yes	2 (10.0%)	15 (15.0%)		2.12 (0.41–11.10)	0.37
No	18 (90.0%)	85 (85.0%)		reference	-
Tumor location			0.51		
Cervical and upper thoracic esophagus	6 (30.0%)	20 (20.0%)		reference	
Middle thoracic esophagus	10 (50.0%)	50 (50.0%)		1.77 (0.49–6.41)	0.38
Lower thoracic esophagus	4 (20.0%)	30 (30.0%)		3.06 (0.64–14.53)	0.16
Macroscopic type			0.89		
0-IIa	4 (20.0%)	16 (16.0%)		-	-
0-IIb	11 (55.0%)	60 (60.0%)		-	-
0-IIc	5 (25.0%)	24 (24.0%)		-	-
Clinical depth of invasion			0.79		
Epithelium/lamina propria	16 (80.0%)	73 (73.0%)		-	-
MM/SM1	3 (15.0%)	19 (19.0%)		-	-
SM2	1 (5.0%)	8 (8.0%)		-	-
Procedure time, min	105.00 ± 62.56	81.53 ± 5.46	0.08	-	-
Endo-knife			0.17		
Hook knife	2 (10.0%)	7 (7.0%)		-	-
IT/Dual knife	18 (90.0%)	84 (84.0%)		-	-
Hybrid knife	0 (0.0%)	9 (9.0%)		-	-
Electrosurgical unit modes			0.64		
Swift coagulation	2 (10.0%)	10 (10.0%)		-	-
Forced coagulation	15 (75.0%)	82 (82.0%)		-	-
Endocut	3 (15.0%)	8 (8.0%)		-	-
Submucosal injection solution			0.04		
Sodium hyaluronate	4 (20.0%)	5 (5.0%)		0.15 (0.03–0.82)	0.03
Other	16 (80.0%)	95 (95.0%)		reference	
Steroids group			0.93		
Oral steroids	9 (45.0%)	44 (44.0%)		reference	
Combined group	11 (55.0%)	56 (56.0%)		0.60 (0.20–1.82)	0.37
Longitudinal length of the resected lesion, mm	63.60 ± 21.77	60.37 ± 21.03	0.53	-	-
Circumferential range			0.01		
<5/6 and ≥ 3/4	17 (85.0%)	57 (57.0%)		0.19 (0.05–0.74)	0.02
≥5/6	3 (15.0%)	43 (43.0%)		reference	
En bloc resection			0.91		
Yes	17 (85.0%)	84 (84.0%)		-	-
No	3 (15.0%)	16 (16.0%)		-	-
Fibrin glue			0.12		
Yes	9 (45.0%)	64 (64.0%)		-	-
No	11 (55.0%)	36 (36.0%)		-	-

CI, confidence interval; OR, odds ratio; BMI, body mass index.

### Complications

There were two patients in the control group, and three patients in the oral prednisone group developed wound bleeding, with patients vomiting blood, which improved after endoscopic hemostatic treatment. One patient in the combined group developed postoperative lung infection, which was not perforated by endoscopy, considering that it was caused by mis-aspiration, following anti-infective treatment the patient’s condition was relieved. No other patients experienced adverse events related to ESD or glucocorticoid or endoscopic dilatation.

## Discussion

For early esophageal cancer, ESD has become the preferred treatment method (4). ESD has a high lesion resection rate, which is conducive to more accurate pathological diagnosis after surgery, and compared with surgery, ESD has the characteristics of less injury to patients and faster postoperative recovery. However, ESD resected more than 3/4 of the esophageal mucosa were often prone to postoperative esophageal stenosis, and the stricture rate is often as high as 80–100% (8). As a result, many researchers have studied the treatment of esophageal strictures, but each treatment has some limitations. Self-expanding coated metal stents and biodegradable stents for the prevention of postoperative esophageal strictures after ESD have been successfully reported, but esophageal stents are also associated with the risks of bleeding, perforation, and migration (20). In addition, some scholars have reported good results in applying autologous tissue transplantation and regenerative medicine to prevent esophageal stenosis, but its safety and effectiveness need to be verified by more clinical studies (11, 21).

Although the effectiveness of oral steroids is well recognized, steroids may cause a number of systemic side effects such as osteoporosis, immunosuppression, diabetes and peptic ulcers and infections (16, 22). Yamaguchi et al. first explored the effectiveness of oral prednisone in preventing esophageal strictures after ESD, and the stricture rate was only 5.3% (1/19), which was more effective than local injection (23). Yet the study by Sato et al. found that oral steroids alone was not effective in preventing post-ESD strictures with circumferential mucosal resection (24). It has even been shown that stenosis still occurs even with steroids prophylaxis in patients with total circumferential resection of the esophagus (14). In our study, the stenosis rate in the oral steroids alone group was 83% (44/53), which was statistically different from the control group. Moreover, in patients with circumferential or near circumferential resection, the stenosis rate was 92.5% (49/53), and not all stenosis occurred, which suggests that oral steroids have a role in preventing stenosis. Although there is still controversy regarding the timing and dosage of oral hormones after esophageal ESD, the most experienced treatment in clinical use is the 8-week regimen, which has been shown to be safer and more effective in several high-quality clinical studies (22–24).

Injection of TA has also achieved good results, but local injection may injure the muscularis propria resulting in complications such as delayed perforation, limiting its widespread use (14). What’s more, local steroid injections have limited effectiveness in circumferential resection (25). The study by Chu et al. combined oral and local steroid injections to investigate their effectiveness in preventing post-ESD strictures, and the rate of strictures was reduced to 14.7% (5/34) in the combined group compared to the control group (52.8%, 19/36) (26). In our study, the stenosis rate was statistically significant (*p* < 0.05) with a decrease in the oral group 44/53 (83.0%) and in the combined group 56/67 (83.6%) compared to the control group 83/87 (95.4%). The reasons behind these differences may be that Chu et al. included patients with resected lesions ≥2/3 of the circumference of the lesion, whereas the present study included patients with resected lesions ≥3/4 of the circumference of the lesion, and that Chu et al.’s study included fewer patients and may have been biased. However, the difference between the oral and combined groups was not statistically significant (*p* = 0.93). Although the combined group could not reduce the stenosis rate compared with the oral steroids group in our study, it was able to reduce the number of dilatations and prolong the interval between the first endoscopic dilatations after ESD, which bought time for the patients to recover from ESD, and to a certain extent, it was conducive to the improvement of the patients’ postoperative quality of life.

With regard to the safety of steroids prophylaxis, a possible complication of local injection of steroids is perforation. Yamashina has reported a case of delayed perforation after local injection of TA, probably due to injury to the lamina propria of the esophagus (27). In addition, oral steroids may cause complications such as osteoporosis, immunosuppression, diabetes, peptic ulcers and infections (16, 22). In our study, the cumulative dose of oral prednisone was 1,120 mg over a period of 8 weeks and no adverse time associated with oral prednisone occurred. However, the study by Waljee et al. noted that even short-term use of steroids increased the risk of adverse events over a 3-year period (28). Therefore, the timing and dosage of oral steroids after esophageal ESD remains controversial and requires further study.

Since steroids have a role in preventing stenosis, what factors influence the occurrence of stenosis after steroids prophylaxis need to be emphasized. Previous studies have shown that spread of the circumference of a resected lesion to seven-eighths or five-sixths of its circumference is a harbinger of stenosis formation (17, 29). This is consistent with the present study, where our study also found that lesion circumference greater than or equal to 5/6 was an independent predictor for the development of esophageal stricture. A study by Wang J et al. noted that longitudinal length of the resected lesion >70 mm was found to be an independent risk factor for esophageal stricture after ESD. The larger the resection lesion, which means the longer the lesion healing time, the higher the likelihood of esophageal stricture (30). Previous studies have shown that lesions in the cervical esophagus are predictors of strictures after endoscopic resection and that strictures in the cervical esophagus are also refractory to dilatation therapy (19, 31). The study by Miyake M et al. also illustrated a history of CRT as a predictor of post-ESD stricture (19). Interestingly, our study showed no statistically significant difference in lesion location in the analysis of stenosis predictors, which is inconsistent with previous studies. This may be due to the small sample size of only 10 patients with cervical and upper thoracic esophageal lesions in our study. Furthermore, our study found that submucosal injection of sodium hyaluronate may be an independent protective factor for stenosis. This may be due to the ability of sodium hyaluronate to better elevate the lesion, providing an adequate safety margin between the mucosal and muscular layers (32). This safety margin may lower hemorrhage and perforation risk peculiar to ESD.

There are some limitations to this study. First, this was a single-center, retrospective study with possible analytical and selection bias. Second, there were statistically significant differences in lesion circumferential size, lesion length, and lesion depth in the baseline data, which may be due to the general belief that steroids have a stenosis-preventing effect, and therefore steroid therapy was preferred for larger and deeper lesions (33). However, some scholars believe that the difference in baseline data is not clinically significant (34). Of course, it may also be caused by the insufficiently large sample size of this study. Third, the control group of patients in this study did not take any measures to prevent stenosis after ESD, mainly because of uncertainty about the efficacy and safety of steroids in early practice. And all patient treatments were individualized and based on the principle of shared decision-making without a standard protocol.

In conclusion, oral prednisone or oral prednisone combined with local injection of TA can effectively prevent esophageal stenosis, reduce the number of endoscopic dilatations and prolong the interval between the first dilation after ESD. Moreover, oral steroid combined with local injection of TA was more advantageous than oral steroid alone in reducing the number of dilatations and prolonging the interval to the first dilatation, which was favorable to the quality of life of patients. In addition, probably advantage of this study was to clarify risk factors of stricture even with steroid administration. Post-ESD esophageal strictures were more likely to occur in patients with a circumference of the resected lesion ≥5/6 and in patients with endoscopic submucosal injection solution without sodium hyaluronate. This opinion provides new insights for further clinical experimental studies to develop more effective methods.

## Author’s note

All authors confirm that the following manuscript is a transparent and honest account of the reported research. This research is related to a previous study by the same authors titled “Predictors of stricture after endoscopic submucosal dissection of the esophagus and steroids application” (35). The previous study was performed on risk factors of developing esophageal strictures in patients using steroids prophylaxis after ESD, and the current submission is focusing on the effectiveness of steroids prophylaxis. The study is following the methodology explained in section of materials and methods.

## Data Availability

The original contributions presented in the study are included in the article/supplementary material, further inquiries can be directed to the corresponding author.
